# Interaction between glutathione S‐transferase M1‐null/present polymorphism and adjuvant chemotherapy influences the survival of breast cancer

**DOI:** 10.1002/cam4.1567

**Published:** 2018-07-21

**Authors:** Shuang Li, Guan‐Tian Lang, Ying‐Zhou Zhang, Ke‐Da Yu, Zhi‐Ming Shao, Qiang Zhang

**Affiliations:** ^1^ Department of Breast Surgery Liaoning Cancer Hospital and Institute Shenyang Liaoning China; ^2^ Department of Breast Surgery Fudan University Shanghai Cancer Center and Cancer Institute Shanghai China; ^3^ Department of Breast Surgery Handan Central Hospital Hebei China

**Keywords:** adjuvant chemotherapy, breast cancer, *GSTM1*, polymorphism

## Abstract

Glutathione S‐transferase M (GSTM) family is concerned with oxidative stress, which is associated with breast carcinogenesis and chemotherapy response. The null polymorphism of *GSTM1* gene results in a thorough absence of the enzyme function. Our study was to evaluate the association between *GSTM1* null/present polymorphism and chemotherapy treatment outcome in breast cancer patients. A total of unrelated 714 patients with a histologically confirmed breast cancer were randomly selected from two independent cancer centers. Polymerase chain reaction was performed to analyze null/present genotypes of *GSTM1* in our study. Our study found that the present genotype of *GSTM1* was associated with a better relapse‐free survival (RFS) (*P *=* *.03) with adjusted hazard ratio (HR) [95% confidence interval (CI)] of 0.63 (95% CI: 0.42‐0.93). The present genotype of *GSTM1* was significantly correlated with a better RFS compared with the null genotype in the nonchemotherapy group (HR = 0.17, 95% CI: 0.06‐0.50; *P *=* *0.001), but no effect was observed in the chemotherapy group (HR = 0.81, 95% CI: 0.52‐1.26; *P *=* *0.35). Moreover, the interaction between the *GSTM1‐*null/present genotype and adjuvant chemotherapy was significant (*P *=* *0.04) in further analysis. Our study suggests that the *GSTM1* polymorphism plays a complex role in influencing the chemotherapy response and breast cancer survival. It is suggested that the *GSTM1*‐present genotype might prevent progression in breast cancer patients. In the meanwhile, it could damage the benefit of adjuvant chemotherapy as well in certain ways.

## 1 INTRODUCTION

It is well‐known that internal estrogens and their metabolites have a tight association with carcinogenesis of the breast in human beings. Estrogen‐quinones and their generated oxidative stress play an important role in this process.^1^ Quinone oxidoreductases and glutathione S‐transferases (GSTs) can decrease the content of quinines or semi quinones in tissues.^2^

Glutathione S‐transferases are effective protection against reactive oxygen species (ROS) via conjugating with glutathione.^3^ *GSTM* family belongs to the predominant enzymes in the breast tissue. The genes of the *GSTM* family are arranged in an alignment of the 5′‐*GSTM*4‐M2‐M1‐M5‐M3‐3′ sequence.^4^ The *GSTM1* gene is localized on chromosome 1p13, and the *GSTM1*‐null genotype seems to be susceptible to many cancers as breast, lung, and colon cancers. The *GSTM1*‐null polymorphism results in a complete absence of *GSTM1* enzyme function which draws us special attention. In many aspects, genetic changes have been demonstrated to be correlated with cancer prognosis, while their effects are still not fully understood up to now.^5^, ^6^, ^7^

Breast cancer is the most prevalent malignant disease in women all over the world, and chemotherapy is undoubtedly important in the treatment of locally advanced solid tumors like breast cancers. Here we hypothesize that *GSTM1*‐null/present polymorphism may have an influence on breast cancer progression and chemotherapy treatment response. To our knowledge, many previous studies have reported the association between *GSTM1* and survival of patients with breast cancer, whereas these studies have come to discrepant conclusions.^8^, ^9^, ^10^, ^11^, ^12^, ^13^ The underlying relationship between oxidative stress and breast cancer prognosis is sort of complicated in the current understanding. Generally speaking, the high level of oxidative stress would increase the response to chemotherapy for the reason that chemotherapy carries out its cytotoxic effects via reactive oxygen species and concomitant oxidative stress. On the other hand, some evidence has showed that oxidative stress is involved in tumor invasion and metastasis.^13^, ^14^, ^15^, ^16^ Therefore, to some extent, the factor whether a patient undergoes chemotherapy or not may cause controversial influences of *GSTM1* polymorphism on breast cancer progression and prognosis.

In this study, we evaluated the role of *GSTM1*‐null/present polymorphism in the treatment outcome of patients with breast cancer, and we also investigated whether this effect would be influenced by adjuvant chemotherapy or not.

## 2 MATERIALS AND METHODS

### 2.1 Patients

This study was approved by the Ethical Committee of the Liaoning Cancer Hospital and the Shanghai Cancer Center, and each participant signed an informed consent document.

A peripheral blood sample, and clinicopathologic information and treatment documents were collected for each patient. The survival outcome was followed up for at least 6 months. We totally recruited 730 independent patients who were pathologically confirmed primary breast cancer diagnosis in the Liaoning Cancer Hospital or the Shanghai Cancer Center from June 2007 to January 2009. Patients who were fulfilled with these following criteria were included in our further study: (1) female; (2) unilateral invasive breast cancer; (3) postoperation patients without any evidence of metastasis; (4) patients with complete adjuvant systemic therapy. Among these 730 patients, 16 cases were excluded because of the failure in *GSTM1* genotyping, and the final 714 cases constituted the final analysis group.

It was because tumor grade and KI67 index were not accessible in many cases that we did not include these variables, while other patients' essential characteristics were collected. The status of estrogen receptor (ER), progesterone receptor (PR), and human epidermal growth factor receptor‐2 (HER2) determined by immunohistochemistry (IHC) staining was confirmed by two independent pathologists in the Department of Pathology of the Liaoning Cancer Hospital or the Shanghai Cancer Center. Patients with indeterminate HER2 protein expression took a fluorescent in situ hybridization test for gene amplification examination.

Postoperative recurrence risk in our present study was mainly categorized according to the St. Gallen consensus 2007 for breast cancer treatment. The choice of applying adjuvant chemotherapy to patients depended on the risk category: patients with moderate recurrence risk would undergo FAC/FEC regimen; patients with high risk would receive taxane‐containing regimens including AC‐P, CAF‐T, and TAC. All of the ER/PR‐positive patients were recommended to take tamoxifen or aromatase inhibitors for at least 5 years after surgery.

### 2.2 DNA/RNA preparation, PCR, and PCR‐based allele genotyping

Genomic DNA was gathered from the patients' peripheral blood leukocytes using Gentra's PureGene DNA Purification kit (Gentra Systems, MN, USA) according to the manufacturer's protocol and was kept at −20°C for storage. The null polymorphism of the *GSTM1* gene was analyzed using PCR in accordance with previously described methods.^13^ The *GAPDH* gene was chosen for an internal control in the experiment. The designed PCR primers of *GSTM1* were showed as following:

sense GAACTCCCTGAAAAGCTAAAGC

anti‐sense GTTGGGCTCAAATATACGGTGG

### 2.3 Survival analysis and statistics

The relapse‐free survival (RFS) in our study was defined as the time from the surgery performed to the first recurrence of disease or the diagnosis of contralateral breast cancer. Patients with loss of follow‐up or study end date were considered to be censored in the survival comparison analysis. Survival outcomes were estimated using the Kaplan‐Meier method, and differences were tested using the log‐rank test (univariate analysis). Hazard ratio (HR) and 95% confidence intervals (95% CIs) were determined by the Cox risk proportion model. The Cox risk proportion model (method: enter) was used to carry out a further multivariate analysis. Significant analysis was carried out by Pearson's χ^2^ test. Student's *t* test was used to compare continuous variables between the two cohorts. All *P*‐values were two‐sided, and a *P*‐value of less than .05 was considered statistically significant. All statistical analysis was computerized on SPSS 17.0 (IBM institute, IL, USA) and Stata/SE 14.0 (College Station, TX, USA) software.

## 3 RESULTS

### 3.1 Association of *GSTM1*‐null/present polymorphism with RFS

Basic characteristics of breast cancer patients and distributions of *GSTM1*‐null/present genotypes are shown in Table 1. The four independent clinicopathological characters, including age, lymph node, tumor size, and IHC‐based subtype, presented no significant association with the *GSTM1* null genotype to the present genotype.

**Table 1:** Characteristics of breast cancer patients and distribution of *GSTM1*‐null/present genotype

Phenotype	Number of patients	*GSTM1*‐null/present genotype				*P* a
		Null		Present		
		N	%	N	%	
Age						
≤45 y	248	139	56	109	44	.94
>45 y	466	259	56	207	44	
Lymph node						
Negative	405	228	56	177	44	.73
Positive	309	170	55	139	45	
Size						
≤2 cm	399	229	57	170	43	.32
>2 cm	315	169	54	146	46	
IHC‐based subtype						
HR+HER2−	415	228	55	187	45	.49
HR+HER2+	85	54	64	31	36	
HR‐HER2−	134	72	54	62	46	
HR‐HER2+	80	44	55	36	45	
Chemotherapy						
No	213	119	56	94	44	.96
Yes	501	279	56	222	44	

HR, hormone receptor; IHC, immunohistochemistry.

a χ^2^ tested *P* values for heterogeneity.

We studied the association of *GSTM1*‐null/present polymorphisms with RFS (Table 2) and found *GSTM1*‐null/present polymorphism had a significant association with RFS (*P *=* *.03) in univariate analysis. A further multivariate analysis demonstrated that lymph node status (*P *<* *.001), tumor size (*P *<* *.001), IHC‐based subtype (*P *<* *.001), chemotherapy or not (*P *<* *.001), and *GSTM1*‐null/present polymorphism (*P *=* *.02) were significant independent factors for RFS.

**Table 2:** Univariate and multivariate analysis of risk factor for relapse‐free survival

	Log‐rank *P*	Adjusted HR^a^ (95% CI)	Adjusted *P*
Age			
≤45 y	.35	Ref.	.33
>45 y		1.21 (0.82‐1.79)	
Lymph node			
Negative	<.001	Ref.	<.001
Positive		3.25 (2.03‐5.18)	
Size			
≤2 cm	<.001	Ref.	<.001
>2 cm		2.50 (1.64‐3.82)	
IHC‐based subtype			
HR+HER2−	<.001	Ref.	<.001
HR+HER2+		2.52 (1.44‐4.42)	
HR‐HER2−		3.28 (1.99‐5.40)	
HR‐HER2+		3.79 (2.27‐6.33)	
Chemotherapy			
No	.06	Ref.	<.001
Yes		0.32 (0.18‐0.57)	
*GSTM1*			
Null	.03	Ref.	.02
Present		0.63 (0.42‐0.93)	

CI, confidence interval; HR, hazard ratio.

a Adjusted for age, lymph node status, tumor size, immunohistochemistry‐based subtype, chemotherapy and *GSTM1*‐null/present polymorphism. HR with its 95% CI is calculated by the Cox risk proportion model.

### 3.2 Association of *GSTM1*‐null/present polymorphism with DFS is modified by adjuvant chemotherapy

**Figure 1:**
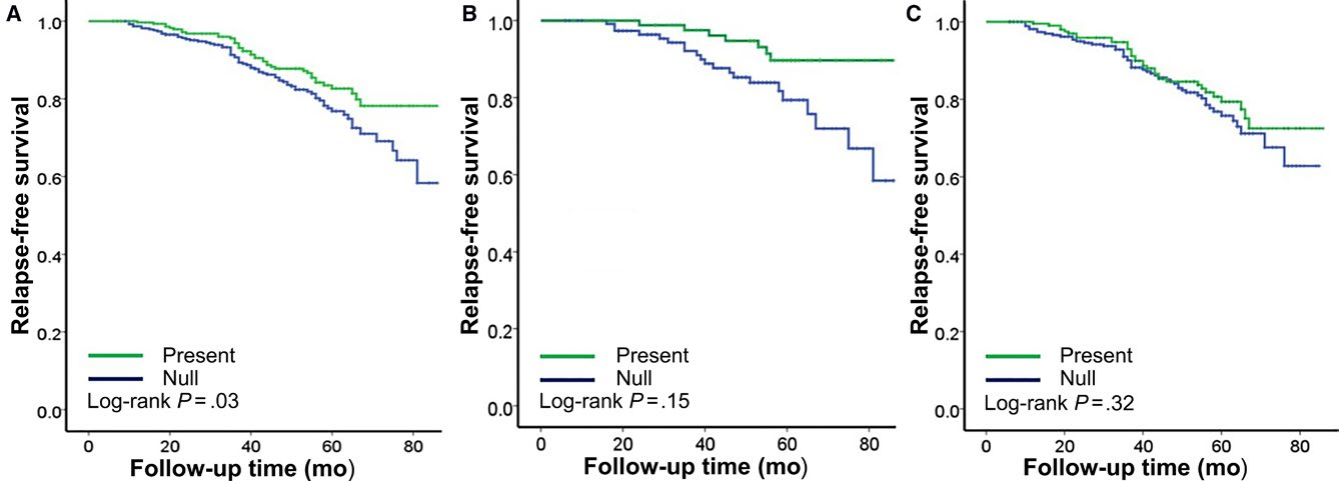
Effects of *GSTM1*‐null/present genotype on relapse‐free survival according to adjuvant chemotherapy in primary breast cancer. A, overall population; B, no chemotherapy; C, with chemotherapy. *P*‐value tested by log‐rank test

We further stratified the patients by age, lymph node status, tumor size, and adjuvant chemotherapy (Table 3). Interestingly, in patients without adjuvant chemotherapy, the unadjusted survival curves showed a statistically significant result (*P *=* *.015, Table 3 **,** Figure 1B). By contrast, in those patients who were treated with adjuvant chemotherapy, the *GSTM1*‐null/present polymorphism had no effect on RFS (*P *=* *.32, Table 3, Figure 1C). The findings presented above strongly suggested the presence of an interaction. After adjustment, the present of *GSTM1* was significantly correlated with better RFS compared with the null genotype in the nonchemotherapy group (HR = 0.17, 95% CI: 0.06‐0.50, *P *=* *.001), but this effect was not preserved in the adjuvant chemotherapy group (HR = 0.81, 95% CI: 0.52‐1.26, *P *=* *.35), with a significant *P‐*value of .04 for interaction between the *GSTM1* genotype and adjuvant chemotherapy.

**Table 3:** Impact of *GSTM1*‐null/present genotype on relapse‐free survival by stratification

	*GSTM1* genotype	Log‐rank *P*	Adjusted HR^a^ (95% CI)	Adjusted *P*	Interaction *P*
Age					
≤45 y	Null	.51	Ref.	.414	>.05
	Present		0.78 (0.43‐1.41)		
>45 y	Null	.03	Ref.	.013	
	Present		0.52 (0.31‐0.87)		
Lymph node					
Negative	Null	.01	Ref.	.013	>.05
	Present		0.40 (0.19‐0.82)		
Positive	Null	.50	Ref.	.232	
	Present		0.75 (0.46‐1.20)		
Size					
≤2 cm	Null	.02	Ref.	.019	>.05
	Present		0.45 (0.23‐0.88)		
>2 cm	Null	.32	Ref.	.247	
	Present		0.75 (0.46‐1.22)		
Chemotherapy					
No	Null	.02	Ref.	.001	.04
	Present		0.17 (0.06‐0.50)		
Yes	Null	.32	Ref.	.35	
	Present		0.81 (0.52‐1.26)		

CI, confidence interval; HR, hazard ratio.

a Adjusted for age, lymph node status, tumor size, immunohistochemistry‐based subtype, and chemotherapy. HR with its 95% CI is calculated by the Cox risk proportion model.

## 4 DISCUSSION

The GST super‐family of enzymes play a vital role in the metabolism of xenobiotics and drugs, including chemotherapeutic agents in breast cancer treatment, like doxorubicin/epirubicin and paclitaxel/docetaxel. The role of *GSTM1* in chemotherapy efficacy and cancer prognosis draws much attention. However, the association between breast cancer disease outcomes and genotypes of *GSTM1* in prior studies still remains inconsistent results.^8^, ^9^, ^10^, ^11^, ^12^, ^13^ In the present study, we mainly investigate the association between RFS and *GSTM1*‐null/present polymorphisms in breast cancer patients. Interestingly, after the adjustment of clinical phenotypes, we find *GSTM1*‐null/present genotypes to be an independent prognostic factor in patients without adjuvant chemotherapy, but not in those with adjuvant chemotherapy.

In previous literature, some observational studies concluded controversial results of the association between the *GSTM1* genotype and treatment outcome in breast cancer patients. Petros et al^17^ reported the median overall survival for the patients with *GSTM1*‐null genotype was significantly longer when comparing to the patients with *GSTM1*‐present genotype. Ambrosone et al^18^ came to a parallel conclusion that women with null genotypes for *GSTM1* and *GSTT1* had reduced hazard of death in relation to those with alleles present. However, Gor et al^19^ observed similar outcomes between *GSTM1*‐null and *GSTM1*‐present patients in their multivariable model. Yang et al^20^ and Duggan et al^10^ also reached a negative result with respect to *GSTM1* genotypes. Our results indicate that *GSTM1*‐present genotype plays protective roles in disease progression if no chemotherapy is administered. However, this effect fades away when the chemotherapy was administrated; that is to say, patients harboring *GSTM1*‐present genotype gain limited benefit from chemotherapy.

The inconsistent survival outcomes between the chemotherapy and nonchemotherapy groups could be explained from three main aspects. First, it is well‐established that the *GSTM1*‐present genotype is associated with elevating activity of estrogen‐quinone metabolizing enzymes, reducing ROS levels and inhibiting oxidative stress (OS)‐induced cancer cell proliferation and angiogenesis. However, most chemotherapy agents exert their cytotoxic effects by elevating the OS levels in the breast carcinoma, increasing OS damage to a level that the cancer cells cannot cope with and leading to cell death.^13^, ^15^ To this point, the originally protective effect of ROS might cause “resistance” to chemotherapy for the *GSTM1*‐present patients. Second, because both anthracyclines and cyclophosphamide are metabolized through reactions mediated by GSTMs,^21^ the presence of *GSTM1* could accelerate the inactivation and metabolism mechanisms of these therapeutic agents and also come to lower levels of circulating active drugs. Third, these different findings might attribute to variation between the studies in different sources of patients, cancer stages, sample collecting ways, or other factors.

Frankly speaking, our study has several limitations. First, genetic variants in other OS‐related genes as well as combinations of these genotypes are not included in the present study. Second, the chemotherapy regimens in our retrospective study are not uniform, and different reactions to specific treatment may be neglected. Third, endocrine therapy for ER/PR‐positive and target therapy for HER2‐positive patients are generally recommended in adjuvant treatment, and their effect on survival is not taken into full consideration in our analysis.

In summary, our results suggest that breast cancer patients with *GSTM1*‐present genotype may gain a better survival outcome when comparing to their wild‐type counterparts, but the benefit probably compromises due to the intervention of adjuvant chemotherapy. The interaction between *GSTM1*‐null/present polymorphism and adjuvant chemotherapy may lead to potential drug resistance and influence the survival of breast cancer patients. The new understanding of interactions between chemotherapy resistance and host genetic factors might contribute to the future design of individualized cancer treatment for patients with breast cancer.

## CONFLICT OF INTEREST

The authors have declared that no competing interests exist.
